# Radioembolization with ^90^Y Resin Microspheres of Neuroendocrine Liver Metastases After Initial Peptide Receptor Radionuclide Therapy

**DOI:** 10.1007/s00270-019-02350-2

**Published:** 2019-10-23

**Authors:** A. J. A. T. Braat, H. Ahmadzadehfar, S. C. Kappadath, C. L. Stothers, A. Frilling, C. M. Deroose, P. Flamen, D. B. Brown, D. Y. Sze, A. Mahvash, M. G. E. H. Lam

**Affiliations:** 1 Department of Radiology and Nuclear Medicine, Imaging Division, University Medical Center Utrecht, Heidelberglaan 100, 3584 CX Utrecht, The Netherlands; 2 Department of Nuclear Medicine, University Hospital Bonn, Bonn, Germany; 3 Department of Imaging Physics, University of Texas MD Anderson Cancer Center, Houston, TX USA; 4 Department of Radiology and Radiologic Sciences, Vanderbilt University, Nashville, TN USA; 5 Department of Surgery and Cancer, Imperial College London, London, UK; 6 Nuclear Medicine, University Hospital Leuven, Leuven, Belgium; 7 Department of Nuclear Medicine, Jules Bordet Institute, Brussels, Belgium; 8 Division of Interventional Radiology, Stanford University, Palo Alto, CA USA; 9 Department of Interventional Radiology, University of Texas MD Anderson Cancer Center, Houston, TX USA

**Keywords:** NEN, Neuroendocrine tumours, PRRT, Radioembolization, SIRT, Yttrium-90

## Abstract

**Purpose:**

Peptide receptor radionuclide therapy (PRRT) and radioembolization are increasingly used in neuroendocrine neoplasms patients. However, concerns have been raised on cumulative hepatotoxicity. The aim of this sub-analysis was to investigate hepatotoxicity of yttrium-90 resin microspheres radioembolization in patients who were previously treated with PRRT.

**Methods:**

Patients treated with radioembolization after systemic radionuclide treatment were retrospectively analysed. Imaging response according to response evaluation criteria in solid tumours (RECIST) v1.1 and clinical response after 3 months were collected. Clinical, biochemical and haematological toxicities according to common terminology criteria for adverse events (CTCAE) v4.03 were also collected. Specifics on prior PRRT, subsequent radioembolization treatments, treatments after radioembolization and overall survival (OS) were collected.

**Results:**

Forty-four patients were included, who underwent a total of 58 radioembolization procedures, of which 55% whole liver treatments, at a median of 353 days after prior PRRT. According to RECIST 1.1, an objective response rate of 16% and disease control rate of 91% were found after 3 months. Clinical response was seen in 65% (15/23) of symptomatic patients after 3 months. Within 3 months, clinical toxicities occurred in 26%. Biochemical and haematological toxicities CTCAE grade 3–4 occurred in ≤ 10%, apart from lymphocytopenia (42%). Radioembolization-related complications occurred in 5% and fatal radioembolization-induced liver disease in 2% (one patient). A median OS of 3.5 years [95% confidence interval 1.8–5.1 years] after radioembolization for the entire study population was found.

**Conclusion:**

Radioembolization after systemic radionuclide treatments is safe, and the occurrence of radioembolization-induced liver disease is rare.

**Level of Evidence:**

4, case series.

## Introduction

With the introduction of radiolabelled somatostatin analogs (SSA), a.k.a. peptide receptor radionuclide therapy (PRRT), especially with ^177^Lu-DOTATATE, treatment of neuroendocrine neoplasms (NEN) has evolved and results in long progression-free survival (PFS) and overall survival (OS). The main accelerator of this development was the recent publication of the NETTER-1 trial, combining ^177^Lu-DOTATATE with long-acting SSA versus high dose SSA alone (control group). ^177^Lu-DOTATATE resulted in a significantly prolonged PFS of 28.4 months compared to 8.5 months in the control group [1, 2]. Because of the long PFS after PRRT in these patients, improving quality of life or postponing deterioration of quality of life becomes even more important, also seen in the NETTER-1 study [3]. Objective response rates according to Response Evaluation Criteria In Solid Tumours (RECIST) version 1.1 assessments were limited, with only 1% complete response (CR) and 17% partial response (PR) [1].

Transarterial radioembolization has gained particular interest in the treatment of liver metastases of neuroendocrine neoplasms (NELM). Radioembolization with yttrium-90 (^90^Y) resin microspheres (SIRSpheres®, Sirtex Medical, Sydney, Australia) has a high intrahepatic success rate and a limited toxicity profile. In patients treated with radioembolization, tumour reduction or stable disease according to RECIST 1.1 occurs in 16% and 75%, respectively [4]. Radioembolization alleviates NEN-related symptoms (like flushing and diarrhoea) in 79% of symptomatic patients [4]. Clinical toxicity is often abdominal discomfort, nausea and fatigue, and is limited to within the first 6 months after treatment. Biochemical and haematological toxicities higher than grade 2 according to the Common Terminology Criteria of Adverse Events version 4.03 (CTCAE) rarely occur (< 7%) [4, 5].

Combining PRRT and radioembolization seems logical in NEN patients with bulky hepatic disease or those with predominant liver tumour burden and extrahepatic disease, since PRRT results in less objective response in bulky liver disease compared to small volume (miliary) liver disease [6]. However, in part based on unpublished anecdotes, concerns have been raised on the potential cumulative hepatotoxicity of PRRT and radioembolization [7, 8]. Hepatotoxicity is suggested to be more prone to occur by combining PRRT and radioembolization; however, evidence and patient specific parameters are lacking. We performed a sub-analysis of our previously reported study, to determine the efficacy and toxicity profile in NEN patients who received radioembolization after PRRT [4].

### Methods

All retrospective data were gathered in the period of July 2015 until October 2016. The inclusion criteria were previously reported [4]: In short, patients with histologically proven NEN (surgical specimen or biopsy), of any origin, with at least baseline and 3 ± 1.5 month follow-up computed tomography (CT) or magnetic resonance imaging (MRI) and previous PRRT were used as inclusion criteria. Details on previous PRRT treatments, clinical complaints and laboratory data were gathered. Baseline characteristics were gathered according to the reporting standards recommended for radioembolization [9].

Prior to the actual radioembolization treatment, all patients received a treatment simulation during a preparatory angiography with technetium-99m macro-aggregated albumin (^99m^Tc-MAA) and followed by a single-photon emission computed tomography (SPECT). Within weeks following the preparatory angiography and imaging, the patient received radioembolization treatment. Ethics approval was obtained according to local regulations at the participating centres.

### Study Outcome Parameters

The primary outcome parameter was hepatic response, according to RECIST 1.1 after 3 months [10]. Secondary outcome parameters included clinical response (improvement of symptoms) and clinical toxicities (adverse events) within 3 months after radioembolization. Biochemical and haematological toxicities at 4–8 weeks and at 3 months were assessed according to the CTCAE version 4.03 [5]. Radioembolization-induced liver disease (REILD) was classified according to terminology defined by Braat et al. [11]. To assess overall survival (OS), date of death or date of last contact (when lost to follow-up) was collected. Because long-term toxicity and OS might be influenced by treatments following radioembolization, additional treatments following radioembolization were collected as well.

### Statistical Analysis

Scatter-plots were made to identify potential correlations between toxicities, and the interval between the last PRRT cycle and radioembolization and cumulative administered PRRT activity. Survival curves were estimated using the Kaplan–Meier method and assessed with the log-rank test. The following variables were tested: radiological response after treatment, tumour grade, intrahepatic tumour load and the presence of extrahepatic disease at time of treatment [4]. *P *values smaller than 0.05 were considered significant in all tests. The database was analysed using IBM SPSS statistics for Windows version 23.0 (IBM, Armonk, NY).

## Results

All patients were treated between December 2006 and May 2016. A total of 58 radioembolization treatments in 44 patients with progressive NELM were included. Baseline characteristics are shown in Table 1. Most patients had diffuse liver metastases with 96% having more than 10 lesions and 93% classified as diffuse, type III pattern [12, 13]. At the time of analysis, 24/44 (54%) patients had died.

**Table 1:** Baseline characteristics at time of first radioembolization (44 patients)

	Years
*Age*	
Mean	59
Range	34-80

	N	%
*Gender*		
Male	24	55
Female	20	45
*Performance score*		
ECOG 0	21	48
ECOG 1	18	41
ECOG 2	5	11
*WHO/ENETS Grade*		
1	13	30
2	22	50
3	3	7
Unknown	6	13
*Primary tumour site*		
Pancreas NF	18	40
Small bowel*	11	25
Large bowel	7	16
Lung/bronchus	3	7
Unknown origin	3	7
Functioning NEN**	2	5
*Extrahepatic metastases*		
No	9	21
Yes	35	79
*Prior treatments*^*†*^		
Surgery	21	48
Somatostatin analogs	28	64
Chemotherapy	16	36
Liver directed therapy	3	7

	%
*Lung shunt fraction*	
Median	3.3
Range	0.9–33^‡^

	GBq
*Administered*^*90*^*Y activity*	
Median	1.7
Range	0.4–5.5

*ECOG* Eastern Cooperative Oncology Group, *WHO/ENETS* World Health Organization/European NeuroEndocrine Tumour Society, *NF* non-functioning.

*Including one gastric NEN

**One gastrinoma and one glucagonoma

^†^Besides the reported systemic radionuclide treatments and radioembolization treatments

^‡^One patient had an LSF exceeding 20% (i.e. 33%), who subsequently received a whole liver treatment in one session without activity reduction and without complications.

### PRRT and Radioembolization Procedure Details

Of the 44 patients, one patient received meta-iodobenzylguanidine monotherapy (^131^I-MIBG; strictly speaking, the molecular target is the norepinephrine transporter, which is a catecholamine pump, and not a peptide receptor, but for the purpose of this study regarded as ‘PRRT’), three patients received ^90^Y-DOTATOC monotherapy, 31 patients received ^177^Lu-DOTATATE monotherapy, and nine patients received a combination of different therapeutic radiopharmaceuticals (Table 2). Median time to radioembolization from diagnosis was 4.6 years (range 1.3–12.9 years), and median time to radioembolization from last cycle of PRRT was 353 days (range 4 days–6.3 years). No extrahepatic depositions of ^99m^Tc-MAA were found on SPECT/CT. Median net administered ^90^Y activity was 1.67 GBq (range 0.4–5.5 GBq), 97% calculated by the body surface area (BSA) method. Most procedures were whole liver treatments (32/58, 55%) (Table 3).

**Table 2:** Systemic radionuclide treatment details prior to radioembolization in 44 patients

Systemic treatment	Number of patients	Number of cycles		Cumulative activity (GBq)	
		Median	Range	Median	Range
^131^I-MIBG only	1	2	NA	14.8	NA
^90^Y-DOTATOC only	3	2	2–5	15.2	10–37
^177^Lu-DOTATATE only	31	4	3–9	30.8	10–61.6
^131^I-MIBG + ^90^Y-DOTATOC	1	1 + 1	NA	11 + 18.6	NA
^90^Y- + ^177^Lu-PRRT	8	2 + 1	1–5 + 1–4	15.2 + 14.8	7.4–32.3 + 7.4–23.3
Total	44	4	2–9	30.4	10–61.6

*NA* not applicable

**Table 3:** Treatment approaches per number of radioembolization treatments (58 procedures in total)

Radioembolization	First	Second	Third	Fourth
One session whole liver treatment	20	2	1	
Sequential whole liver treatment*	9			
Lobar treatment	15	8	2	
Selective treatment				1

*Right lobe first and left lobe second with an interval of 4–6 weeks, or vice versa

### Imaging and Clinical Response

An objective response rate of 16% and disease control rate of 91% were observed at 3 months according to RECIST 1.1 (CR 2%, PR 14%, stable disease 75% and progressive disease 9%). Malignancy-related symptoms were present at treatment in 23/58 (40%) of procedures prior to radioembolization. Abdominal pain (35%) and flushing (30%) were most frequently reported. Clinical response occurred in 15/23 (65%) of these patients after 3 months, with 7/23 (30%) having improvement of pre-treatment complaints and 8/23 (35%) experiencing complete resolution of pre-treatment symptoms after radioembolization. 8/23 (35%) remained symptomatic after radioembolization.

### Toxicity

**Fig. 1:**
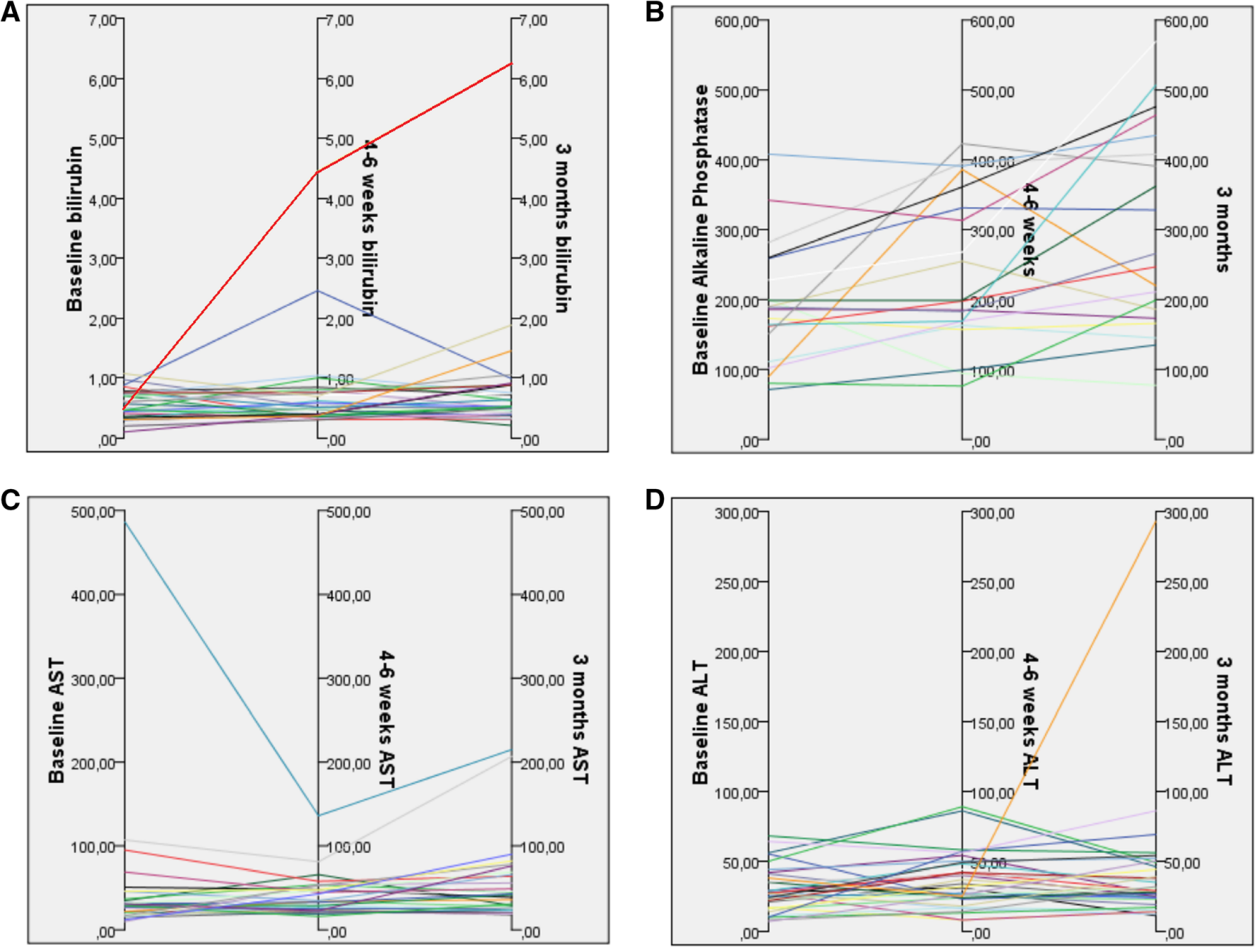
Biochemical toxicities in the first three months after radioembolization. **A** Bilirubin measurements in mg/dl; **B**–**D** Alkaline phosphatase, aspartate aminotransferase (AST) and alanine aminotransferase (ALT) in U/l. One patients developed a grade 5 REILD with deteriorating bilirubin levels (**A**) and increasing ALT values at 3 months (**D**). One patient had isolated AST elevation prior to radioembolization (**C**) potentially related to prior treatment with everolimus

At 3 months after radioembolization, no clinical toxicities occurred in 37/58 (64%), known radioembolization-related adverse events occurred in 15/58 (26%), mainly transient abdominal discomfort, and the treating physician did not register clinical toxicities in 6/58 (10%; missing data or death). At baseline, most patients already had a variety of biochemical toxicities according to CTCAE v4.03 (Table 4). The most common newly developed CTCAE grade 3–4 biochemical and haematological toxicities were γ-glutamyl transpeptidase (γGT) elevation (10%) and lymphocytopenia (42%). New grade 3 hyperbilirubinemia occurred in one patient who developed REILD (Fig. 1A). Dynamics in bilirubin, ALP, AST and ALT after radioembolization are depicted in Fig. 1. In the scatter-plot analyses, no correlation was found between toxicities, cumulative PRRT activity and the interval between PRRT and radioembolization.

**Table 4:** Absolute percentages of biochemical and haematological toxicities

CTCAE grade*	Baseline			4–6 weeks			3 months		
	0 (%)	1 + 2 (%)	3 + 4 (%)	0 (%)	1 + 2 (%)	3 + 4 (%)	0 (%)	1 + 2 (%)	3 + 4 (%)
ALP	47	51	2	25	75		25	75	
γGT	15	47	38	5	41	54	3	31	66
AST	75	23	2	64	36		55	45	
ALT	79	21		78	22		76	22	2
Bilirubin	95	5		90	8	3	87	10	3
Albumin	73	27		64	36		55	45	
LDH	61	37	2	56	44		69	28	3
Haemoglobin	27	73		29	71		25	75	
Leucocytes	75	25		77	23		75	23	2
Lymphocytes	45	32	23	18	54	26	25	33	42
Platelets	82	17	2	69	29	2	70	28	3
INR	100			100			100		

*CTCAE* Common terminology criteria for adverse events version 4.03, *ALP* alkaline phosphatase, *γGT* γ-glutamyl transpeptidase, *AST* aspartate aminotransferase, *ALT* alanine aminotransferase, *LDH* lactate dehydrogenase and *INR* International Normalized Ratio

### Radioembolization-Related Toxicities

Radiation-induced gastric ulceration occurred in two patients (5%), both confirmed by endoscopy (one biopsy proven). Radiation pneumonitis occurred in one patient (2%). One patient developed a liver abscess (2%), and one patient developed cholangitis (2%).

Two patients developed REILD as reported by the treating physician; the first patient was previously treated with seven cycles of ^177^Lu-DOTATATE (55.4 GBq). Whole liver radioembolization in one session with 2.7 GBq was performed 4.6 years after the last cycle of ^177^Lu-DOTATATE. The patient developed ascites without significant biochemical toxicities and ascites decreased without medical intervention, and thus this was retrospectively classified as grade 2 REILD. Six months after radioembolization, extrahepatic disease progressed and the patient received additional ^177^Lu-DOTATATE (5.5 GBq), without evidence of REILD.

The second patient was heavily pre-treated and had three cycles of ^177^Lu-DOTATATE (20.1 GBq). Sequential whole liver radioembolization (right lobe first and left lobe six weeks later) with a cumulative activity of 5.0 GBq (partition model calculation) was performed 3.2 years after the last cycle of ^177^Lu-DOTATATE. The patient developed abdominal discomfort, ascites, CTCAE grade 3 hyperbilirubinemia and CTCAE grade 2 ALP elevation. Toxicities persisted till the patient died 20 weeks after radioembolization (REILD grade 5).

In retrospect, one other patient developed clinical and biochemical evidence of REILD, but was not reported by the treating physician. The patient had grade 3 REILD, based on grade 2 bilirubin elevation after 4 weeks (at baseline already grade 1) and development of ascites, without evidence of tumour progression. Bilirubin levels returned to grade 1 within 3 months (Fig. 1A) and ascites resolved with additional diuretics (spironolactone and furosemide).

### Treatments after both PRRT and Radioembolization

A total of 34/44 patients (77%) received additional treatment after PRRT and radioembolization, apart from the reported additional radioembolization procedures in 10 patients (Table 3). Long-acting SSA therapy was continued in 18/44 patients (41%). In 19/44 patients (43%) additional PRRT treatments were given with ^177^Lu-DOTATATE with a median of one treatment cycle (range 1–7 cycles) and a median cumulative activity of 9.2 GBq (range 5.5–41.5 GBq). Other treatments were less common and consisted of systemic chemotherapy (27%), surgery (9%) or additional liver-directed therapies (9%; bland-embolization and radiofrequency ablation).

### Overall Survival

Median OS after radioembolization for the entire population was 3.5 years; range: 51 days (lost to follow-up)–7.6 years [95% CI 1.8–5.1 years]. Median OS for grade 1 NEN was 3.6 years [95% CI 2.7–4.3]. Median OS for grade 2 NEN was 2.8 years [95% CI 0.6–4.6], and for grade 3 NEN was 136 days (range 115–504 days). Patients with an unknown tumour grade had a median OS of 262 days (range 73–644 days).

**Fig. 2:**
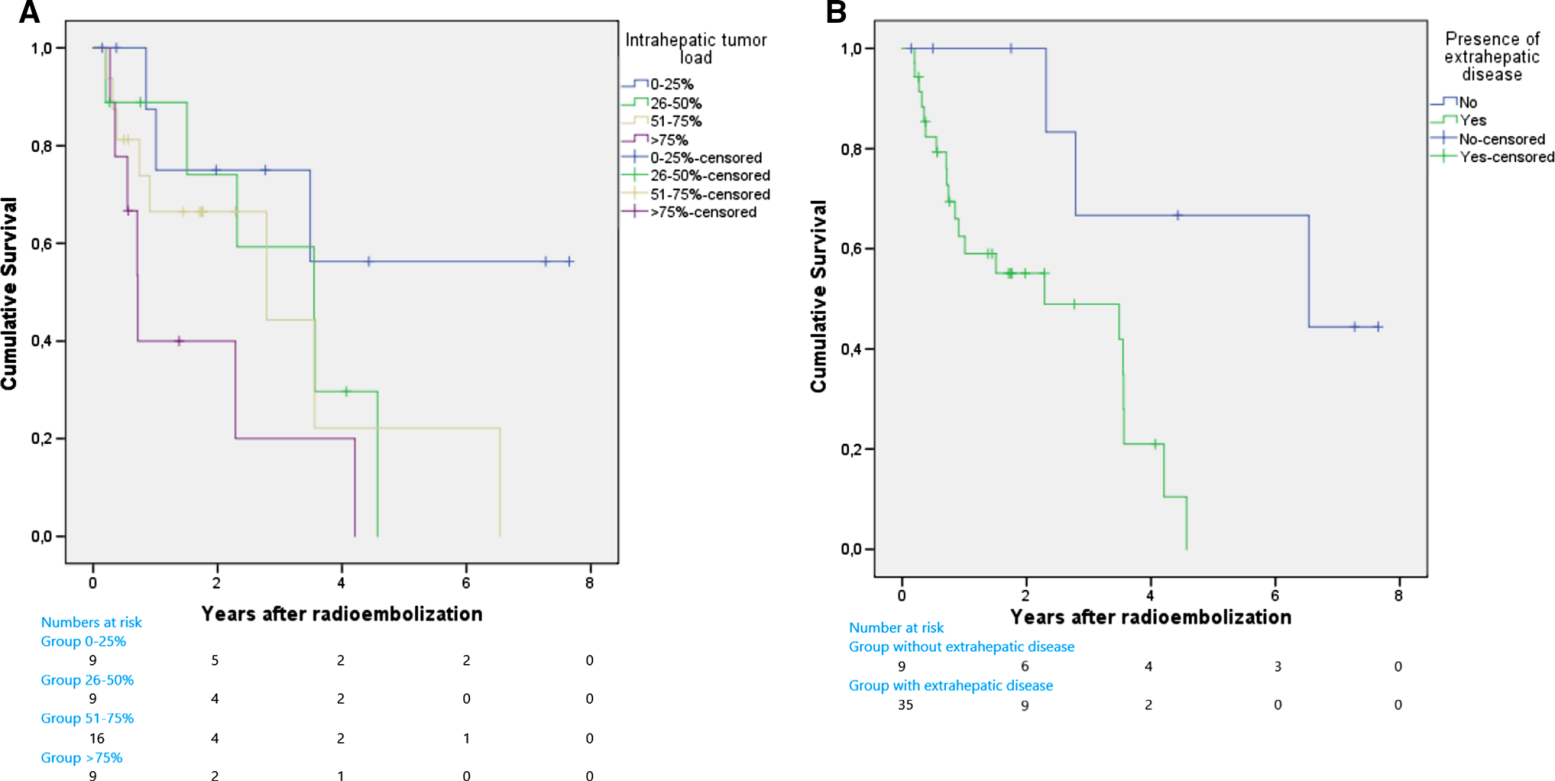
Kaplan–Meier survival curves of two factors with a negative impact on overall survival. **A** Intrahepatic tumour load. Patients with > 75% tumour load have a significant shorter overall survival compared to patients with < 75% tumour load (*p* = 0.007). **B** Presence of extrahepatic disease at time of radioembolization. Patients with extrahepatic disease have a significant shorter overall survival compared to patients without extrahepatic disease (*p* = 0.001)

Kaplan–Meier analyses confirmed intrahepatic tumour load > 75% as a significant negative prognostic factors for OS (*p* = 0.007; Fig. 2A). Presence of extrahepatic disease resulted in a poorer OS as well; median OS 3.2 years [95% CI 1.1–5.3] versus 6.2 years [95% CI 5.5–7.0] (*p* = 0.001; Fig. 2B). In the Kaplan–Meier analyses, OS was independent of disease control rate (*p* = 0.7) or objective response rate (*p* = 0.7) according to RECIST 1.1.

## Discussion

In this study, radioembolization of progressive NELM after initial PRRT resulted in a disease control rate of 91% at 3 months according to RECIST 1.1, clinical response in 65% of symptomatic patients and a long median OS of 3.4 years (41 months). Intrahepatic tumour load > 75%, and the presence of extrahepatic disease prior to treatment were negative prognostic markers for OS [4]. Occurrence of REILD after treatment was limited (5%). Our data showed no correlation between treatment toxicities and the time interval between PRRT and radioembolization, and cumulative activity of previous PRRT.

Previously reported studies on hepatotoxicity after radionuclide treatments are limited, are all retrospective studies, consist of small populations, and are difficult to interpret. Looking at hepatotoxicity following PRRT, the NETTER-1 study reported no hepatotoxicity [1]. However in 2015, Riff et al. reported an increased hepatotoxicity rate after ^90^Y-PRRT (n = 17) compared to standard-of-care (n = 76) and the authors suggested that prior radioembolization treatment increased the likelihood of hepatotoxicity, even though the correlation was non-significant [7]. More recently in 2017, Su et al. described long-term hepatotoxicity in 54 patients after whole liver and lobar radioembolization with ^90^Y glass microspheres in NET patients [8]. In their cohort, hepatic decompensation could be attributed solely to the radioembolization in just 2 patients. To our knowledge, just one study discusses the occurrence of hepatotoxicity in NEN after radioembolization with ^90^Y resin microspheres. Tomozawa et al. reported their findings in 52 patients with more than 1-year follow-up [14]. None of the patients had prior systemic radionuclide treatments or whole liver treatments in one session, and 29 out of 52 patients received bilobar treatment. Mainly CTCAE grade 2 biochemical toxicities were found and CTCAE grade 3 biochemical toxicities were found in just 8%. Cirrhosis-like morphology or portal hypertension on imaging was found in 15 patients (29%). However, other treatments following radioembolization were not reported and influence of other (hepatotoxic) treatments remains to be determined [14].

In 2012, Ezziddin et al. reported on the safety of radioembolization after previous ^177^Lu-DOTATATE treatment. In 23 patients a limited number of adverse events < 10% (CTCAE version 3.03 grade 3 or 4) was reported with an objective response rate of 30% (after 3 months according to RECIST 1.0) and long OS of 29 months following radioembolization [6]. These results seem quite comparable to our results. However, 35% of the cohort reported by Ezziddin et al. did develop a CTCAE v3.03 grade 1 or 2 ascites, which is a higher occurrence of ascites compared to our data (5%). Unfortunately, both the cumulative administered activity of ^177^Lu-DOTATATE and the time interval between ^177^Lu-DOTATATE and radioembolization were not reported.

Two major issues currently exist in the literature. First, there is no accepted standardized definition for radiation-induced hepatotoxicity. Especially, since the adoption of radioembolization, the definition of REILD in the literature has been vague and variable. We recently proposed a new classification system to define REILD, which could be applied to all patients, in line with the CTCAE system [11]. However, this proposed system has yet to be adopted and differs from the definitions reported in the other studies. Lack of standardized definition of hepatotoxicity prevents valid comparison of studies.

Second, there is no established quantitative relationship between radiation absorbed dose in healthy liver tissue and hepatotoxicity for radionuclide therapies. There are no conclusive dosimetric data to support this theory, and neither PRRT nor radioembolization has validated voxel-based dosimetric methods available. The previously mentioned studies only suggested this phenomenon, without providing quantitative dosimetry.

Besides the relatively short follow-up period and retrospective nature of our study, the lack of dosimetric data is the main limitation of our study. A dose–response correlation was reported in other tumour types, including hepatocellular carcinoma and colorectal carcinoma, which may be regarded as a call for additional research on dosimetry in NEN [15, 16]. Unfortunately, not all recruiting centres acquired post-treatment ^90^Y-bremsstrahlung SPECT or ^90^Y-PET imaging, making post-treatment dosimetric evaluation impossible. However, our study does describe the longitudinal medical history of the treated patients. Even after a combination of PRRT and radioembolization, there seems to be room for additional radioembolization treatments (Table 3) or even ^177^Lu-DOTATATE. This also emphasizes the need for accurate dosimetric data on radioembolization and PRRT in NEN patients in future studies, not only looking at tumour absorbed dose and objective response, but also at healthy tissue absorbed dose and hepatotoxicity.

Furthermore, PFS could not be measured reliably in this retrospective series since follow-up imaging intervals were not standardized across all centres. Follow-up was limited to 3 months after treatment in this cohort as most patients went on to receive subsequent treatment, even before intrahepatic PD was documented according to RECIST 1.1, while some other patients were lost to follow-up after the response assessment. Patients lost to follow-up and subsequent treatments prior to intrahepatic PD after radioembolization made imaging and toxicity follow-up beyond 3 months unreliable.

Besides the need for accurate dosimetric data in radioembolization, prospective randomized controlled studies in NEN are clearly needed. The sequencing of radioembolization amongst other treatment options deserves to be studied in carefully designed prospective trials.

## Conclusion

Radioembolization after systemic radionuclide treatments is safe, and the occurrence of radioembolization-induced liver disease is rare.
